# Development and Validation of the Emergency Department Transitions Measure

**DOI:** 10.1111/acem.70252

**Published:** 2026-03-17

**Authors:** Sara Beachy, Danielle M. McCarthy, Julianna Lenoir, Alexzandra Gentsch, Richard Hass, Marissa Witmer, Paula Ostroff, Melissa Tupas, Lindsey Shughart, Hailey Shughart, Kristin L. Rising

**Affiliations:** ^1^ Sidney Kimmel Medical College Thomas Jefferson University Philadelphia Pennsylvania USA; ^2^ Feinberg School of Medicine Northwestern University Philadelphia Pennsylvania USA; ^3^ Center for Connected Care Thomas Jefferson University Philadelphia Pennsylvania USA; ^4^ College of Population Health Thomas Jefferson University Philadelphia Pennsylvania USA; ^5^ West Virginia School of Osteopathic Medicine Lewisburg West Virginia USA; ^6^ Lewis Katz School of Medicine Philadelphia Pennsylvania USA

## Abstract

**Background:**

Transitions of care are high‐risk periods for patient safety in the emergency department (ED), particularly for patients who are still in the diagnostic process and are discharged with diagnostic uncertainty. Care transitions must be improved for these patients, as one third of discharged ED patients have diagnostic uncertainty. Yet there are no validated measures that assess the quality of care transitions from the ED, limiting the ability to assess the impact of interventions. Thus, we developed and validated the ED Transitions (EDT) measure.

**Methods:**

This mixed methods study was conducted across a large healthcare system in three phases: item generation, cognitive interviewing, and large‐scale validation. Scale items were generated by experts and then iteratively refined using feedback from cognitive interviews (*n* = 11). The measure was then validated on a large sample of patients (*n* = 301) recently discharged from the ED. Exploratory structural equation modeling (ESEM) was employed to assess factor structure. Bivariate correlations were used to assess discriminant and convergent validity using the Care Transition Measure (CTM‐3) and the Communication Assessment Tool‐Teams (CAT‐T).

**Results:**

The measure was iteratively refined by way of an expert panel and cognitive interviews which resulted in a 15‐item measure to be used for validation. The validation sample (*n* = 301) was 62% women, 49% White, and the majority having Medicare and/or Medicaid (68%). Sequential comparisons between confirmatory factor analyses and ESEM resulted in a final 10‐item two‐factor structure. Reliability was excellent (0.93), and bivariate correlations indicated positive correlations between the EDT, CTM‐3, and CAT‐T.

**Conclusion:**

The EDT measure demonstrates content validity, structural validity, convergent validity, discriminant validity, and high internal consistency (i.e., reliability). This newly developed patient reported outcome measure can be used in future clinical and research work to better understand the impact of ED interventions on quality‐of‐care transitions for patients with diagnostic uncertainty.

## 1 Introduction

Transitions of care are high‐risk periods for patient safety, particularly when patients are discharged with diagnostic uncertainty and thus are still within the diagnostic process [1, 2, 3, 4, 5, 6, 7, 8]. Effective communication between clinicians and patients is essential for high quality care transitions [9, 10, 11]. Communication of discharge diagnosis, prognosis, treatment plan, and expected course of illness are key characteristics of high‐quality emergency department (ED) discharge [12]. For patients discharged from the ED with diagnostic uncertainty, however, there are often no standardized instructions to communicate. As a result, patients often leave the ED with unaddressed fear related to their symptoms and face a troubling dichotomy. If they are unaware of the uncertainty, they may not follow up appropriately or may ignore dangerous symptoms. Alternately, their fear may cause heightened sensitivity to their symptoms and lead them to seek unnecessary care.

The nature and limitations of emergency care require a focus on real‐time identification of life‐threatening conditions. Thus, while there is opportunity to increase diagnostic accuracy in the ED, realistically, unless our core function as emergency providers changes, there will always remain a significant proportion of patients discharged with a preliminary or symptom‐based diagnosis. Yet, there are no existing validated measures to assess quality of care transitions home from the ED that are inclusive of patients with diagnostic uncertainty. Further, there are few available measures for assessing care transitions from the ED more generally, with existing measures either originally not designed for the ED context or designed to focus on specific populations [13, 14]. This measurement gap limits the conduct of future high‐quality observational and interventional trials to improve care transitions home for patients with uncertainty. This is especially imperative as symptom‐based discharge diagnoses account for approximately 38% of all ED discharges [15, 16]. Further, few ED‐based care transition interventions effectively reduce 30‐day ED return visits, suggesting the need for increased research so more targeted, efficacious interventions can be created. A validated measure that assesses patients' perceptions of quality‐of‐care transitions, particularly when diagnostic uncertainty exists, is a critical next step.

To that end, in the following we report on our work to develop and validate the Emergency Department Transitions (EDT) measure, a patient‐reported outcome measure designed to assess the quality‐of‐care transitions home from the ED for patients with diagnostic uncertainty. Initial development and validation work for the EDT was in the use‐case of patients with diagnostic uncertainty, thus facilitating conduct of future trials focused on care transitions for this high‐risk population. Subsequent work may allow for validation of this measure in other patient populations for use more broadly as an ED‐focused care transitions measure.

## 2 Methods

### 2.1 Study Design

This mixed methods study was conducted at Thomas Jefferson University and at affiliated hospitals within the Jefferson Health enterprise from January 1 to September 24, 2024. At the time of the study, Jefferson Health comprised a 17‐hospital academic health system spanning nine counties and two states. As our goal was to develop a patient‐reported outcome measure, we followed measure development guidelines outlined for the field of medicine and the Patient‐Reported Outcomes Measurement Information System (PROMIS) [17, 18]. In the first phase, we developed and refined an initial draft of the tool in collaboration with an expert panel. In the second phase, we refined the draft through cognitive interviews with patients with a focus on ensuring tool items were comprehensive, appropriate, and useful. In the final phase, we tested the reliability and validity of the EDT as well as the scale structure across a diverse sample of patients discharged with diagnostic uncertainty from across 12 EDs within the Jefferson Health system. We only recruited from these 12 hospitals, and the remaining hospitals were excluded due to the following reasons: (1) did not use the same electronic health record software and/or (2) the hospital did not have an ED. All study activities were approved by the Thomas Jefferson University Institutional Review Board. All patients provided verbal informed consent and were compensated $40 for study participation.

### 2.2 Participants and Inclusion Criteria

Across all phases involving patient‐recruitment, inclusion criteria were met if patients: (1) were 18 years or older, (2) were seen in a Jefferson ED and planned for discharge from the ED or discharged from the Jefferson ED within the past 2 days, (3) were able to provide informed consent, (4) spoke English, and (5) were assigned a symptom‐based diagnosis in the EHR clinical impression. For a symptom‐based diagnosis, research coordinators were provided a list generated by two ED physicians who were part of the study team. This list (Appendix A) included but was not limited to chest pain, abdominal pain, and shortness of breath. If any research coordinators needed clarification on if the patient's diagnosis was symptom‐based, they consulted with the study physicians. Exclusion criteria included patients who were (1) admitted to the hospital, (2) clinically unstable, psychologically impaired or intoxicated, and (3) unable to provide informed consent.

### 2.3 Study Phases

#### 2.3.1 Phase 1: EDT Draft Development

Development of the EDT was grounded in prior work of our team, in which we extensively engaged patients to explore their needs related to seeking a diagnosis in the ED as well as at the time of discharge home from the ED [19, 20]. We worked internally to generate an initial version of the EDT based on items across three tools (see Appendix B for more details):

- Uncertainty Scale (U‐Scale): The 30‐item U‐Scale was developed by our team in prior work as a means of quantifying patient uncertainty at the time of ED discharge [21, 22, 23]. It contains seven subdomains (e.g., treatment quality, diagnosis, self‐management, decision to seek care).
- Uncertainty Communication Checklist (UCC): The UCC was developed by our team as a conversational approach to promote more effective ED discharge conversations between patients and clinicians when patients are discharged with diagnostic uncertainty [20, 24, 25]. The UCC is clinician facing but was designed in close collaboration with patients to reflect their needs at time of discharge from the ED.
- Short‐Form Care Transitions Measure (CTM‐3): The CTM was developed as a measure to capture the quality of hospital‐to‐home care transitions to aid quality improvement [26]. Confirmatory factor analysis indicates that the CTM‐3 accounts for 88% of the variance in the 15‐item CTM score (i.e., the CTM‐3 closely approximates the CTM −15). Thus, we utilized the CTM‐3 to help generate items for the EDT measure.

Items from across these measures were sorted into buckets based on similarity to identify key concepts being assessed. These concepts were then grouped by the team into three primary domains: communication, quality of care/trust, and patient self‐efficacy. The team developed items to represent each domain, using language from the previously developed measures when possible. The goal was to develop a patient‐facing measure of patients' understanding of care transitions upon discharge from the ED. We developed the measure iteratively with an expert panel (Appendix C). This process was done to ensure that we comprehensively captured relevant concepts/domains and that the measure had appropriate clinical and research applicability.

#### 2.3.2 Phase 2: EDT Refinement

In this stage, we conducted 11 cognitive interviews with a convenience sample of patients discharged from the ED with diagnostic uncertainty to review the draft EDT. The goal of cognitive interviews was to ensure that the measure had adequate content validity and response process. Content validity refers to the degree to which the scale accurately captures the construct that it was intended to assess. Response process refers to the degree to which a patient understands the items, what is being asked, and the ease at which they can respond. Cognitive interviews are key to determining whether patients perceive the items as being relevant to their experiences and clearly capture the construct that researchers intend to study [27]. Cognitive interviews used a standardized probing approach to ensure that the EDT items were understandable and accurately measured the intended construct of transition of care quality in patients with diagnostic uncertainty [28, 29].

Two team members reviewed daily patient discharges from the ED and called eligible patients to inquire if they were interested in participating in the cognitive interviews. All interviews were conducted within 2 days of their discharge from the ED (duration: January 1, 2024–February 7, 2024). Patients provided verbal informed consent and completed a demographics survey at the time of enrollment. Interviews were audiotaped and reviewed.

#### 2.3.3 Phase 3: EDT Validation Testing

In this final phase, we administered the EDT along with a set of other measures described below to a large sample of patients (*n* = 301) who had recently been discharged from any of the Jefferson Health EDs to test convergent and discriminant validity. Convergent validity refers to the extent to which two or more scales that measure the same or similar constructs are correlated. Discriminant validity refers to whether scales that measure different or unrelated constructs are correlated. Enrolled patients were asked to provide demographic data, complete all EDT questions, and respond to the following four additional surveys:

- *Care Transitions Measure 3‐Item (CTM‐3)*. The CTM‐3 assesses patient perspectives of the quality of care transitions they experience between different healthcare locations and services [8]. The 3‐item CTM‐3 uses a 4‐point scale and measures patient‐reported readiness for hospital discharge as it relates to the discharge process (i.e., provider‐patient communication, instructions). Previous studies have found that lower scores on the CTM‐3 are significantly associated with increased likelihood for returning to the ED within 14 days and treatment nonadherence [13]. As this measure assesses care transitions, it was used to assess convergent validity, with an anticipated strong positive correlation with the EDT.
- *Communication Assessment Tool‐Teams (CAT‐T)*. The 15‐item CAT‐T uses a 5‐point Likert scale and measures patients' satisfaction with medical team‐patient communication and interpersonal skills (e.g., Providers: *Showed care and concern*) [30]. As transitions of care are directly associated with providers' abilities to communicate discharge summaries and instructions clearly, this was used to assess convergent validity, with an anticipated strong positive correlation with the EDT.
- *Spielberger State‐Trait Anxiety Inventory Short Form (STAI‐SF)*. The STAI assesses an individual's appraisal of situations and the degree to which they find the situation threatening or anxiety‐provoking [31]. The 6‐item STAI‐SF, developed from the 40‐item original scale, uses a 4‐point scale and measures transient anxiety. Previous studies have employed the STAI in studies of care received in the ED and have found that the level of anxiety measured on the STAI is correlated with satisfaction with care [32]. As the EDT is intended to comprehensively measure readiness in care transitions from the ED to home as opposed to trait anxiety, this measure was used to assess discriminant validity, with an anticipated nonsignificant correlation.
- *PROMIS companionship*. This 4‐item measure uses a 5‐point Likert scale and assesses companionship in adults (e.g., *Do you have someone with whom to have fun?*). This measure assesses social isolation and is associated with increased hospitalization, cost, and mortality [33]. As this construct is unlikely to have any correlation with the newly developed EDT, this measure was selected to assess discriminant validity.

All participants provided verbal informed consent. Participants answered survey questions by phone and their responses were recorded in REDCap by research staff.

### 2.4 Data Analyses

The Statistical Package for the Social Sciences (SPSS) was used to clean data and conduct descriptive statistics and bivariate correlations. Exploratory structural equation modeling (ESEM) via Mplus v. 8.9 was employed to assess the structure of the EDT measure. ESEM is a statistical method which combines the flexibility of exploratory factor analysis (EFA) with the strengths of confirmatory factor analysis (CFA) (e.g., controlling for measurement error) [34]. An EFA is an exploratory analysis that helps with item reduction and selection in addition to identifying the number of factors that represent the data by identifying patterns of correlations. A CFA is a confirmatory analysis that helps verify if the factor structure and individual items adequately reflect the data based on the strength of correlations each item has on its respective factor. ESEM allows items to load onto multiple factors (as in EFA) while also imposing constraints usually seen in CFA [34].

Unlike ordinary factor models, which treat sub‐factors as distinct and possibly correlated, bifactor models incorporate a general factor influencing all items as well as specific sub‐factors. Based on our previous work, we hypothesized that care transitions reflect a bi‐factor structure that consists of an overall readiness for care transitions in the ED construct along with distinct sub‐components of transitions of care (e.g., perception of quality of care, patient self‐efficacy). ESEM can test bifactor models while a standard CFA would split readiness for care transitions into a combination of individual factors.

Given the complexity of ESEM, best practice dictates that researchers follow a prescribed guideline which begins with an EFA to assess structure followed by a decision‐tree which results in comparisons between ESEM and CFA structures. We followed procedures outlined in Swami and colleagues, Prokofieva and colleagues, and Zyl and Klooster [34, 35, 36]. This iterative process takes a data‐ and theoretical‐driven approach to item reduction and finalizing a measure structure [34]. Further, as this process is sequential and combines EFA and CFA processes, it does not require additional samples unlike traditional measure development studies that only use EFA and CFA. Instead, researchers can utilize the same sample during the sequential evaluation of the measurement models [35, 37]. ESEM is also advantageous to use as factor loadings and correlations of measurement models are used for interpretation instead of statistical significance, eliminating traditional sample size estimation requirements of structural equation modeling [34]. However, to ensure a robust sample, we aimed to recruit 200–400 participants [38].

#### 2.4.1 Sequential Process of ESEM

*EFA*: Prior to conducting the factor analyses, the inter‐item correlation matrix was analyzed to assess both redundancy and cohesiveness of items. An EFA was then conducted to identify the initial structure, and 1‐, 2‐, 3‐, and 4‐factor structures were compared. To identify the best initial structure, several criteria were used including (1) goodness‐of‐fit indices, (2) a significant χ [2] test comparing factor structures, (3) the 40‐30‐20 rule, (4) considerations for local identification, and (5) interpretability and parsimony of the structure to maximize the application of the measure in the real world. Incremental, absolute, and parsimony fit indices were used with a priori specified cutoff values. Values > 0.90 and > 0.95 for the comparative fit index (CFI) and the Tucker‐Lewis model index (TLI) indicated adequate and excellent fit, respectively. A non‐significant χ [2] *p‐*value > 0.05 indicated adequate fit. Values < 0.08 for the root‐mean‐square‐error of approximation (RMSEA) and values < 0.05 for the standardized root‐mean‐square residual (SRMR) indicated adequate fit [39, 40]. We also evaluated χ [2] tests comparing each factor structure to determine which structure had a better fit. In this case, a significant *p* value indicated that the structure with more factors had a better fit for the data. Additionally, we prioritized structures where items had at least a 0.40 correlation on a primary factor but had minimal cross‐loadings (< 0.3). See Appendix D for definitions of the statistical analyses. The oblique rotation, Geomin, was used as factors of care transitions were expected to be correlated.

*CFA and ESEM*: Model fit criteria were assessed using the same methods as the EFA. Thus, a combination of incremental, absolute, and parsimony fit indices (i.e., CFI, TLI, RMSEA, SRMR, Chi‐square) were employed with the same cut‐off values. In alignment with ESEM, a CFA was first conducted and evaluated for model fit using these criteria. After establishing model‐fit, factor loadings and item uniqueness were evaluated to assess the relationship between each item and the latent construct (i.e., perceptions of transitions of care during diagnostic uncertainty). Loadings greater than 0.35 and residual error variances between 0.10 and 0.90 were considered adequate [36]. After establishing measurement quality, ESEM was employed following an iterative decision‐making process [34, 35, 36, 37]. After identifying the best solution in ESEM, goodness‐of‐fit indices were compared between the CFA and ESEM. Per the guidelines, if goodness‐of‐fit indices indicated minimal differences between the two analyses (e.g., CFA and ESEM), a CFA was used to evaluate a bi‐factor structure.

## 3 Results

### 3.1 Study Phase 1: EDT Draft Development Item Generation

After reviewing items from the existing measures, we identified 16 questions that our previous research indicated were salient to the patient experience of the ED discharge and of diagnostic uncertainty. Subsequently, we met with the expert panel which provided feedback on both the content and response scale of the items. Experts recommended the removal of one item, resulting in a total of 15 questions that utilized a 4‐point response scale (i.e., *Strongly Disagree, Disagree, Agree, Strongly Agree*).

### 3.2 Study Phase 2: EDT Refinement

Eleven participants completed cognitive interviews of the EDT. All participants were asked questions (e.g., *Tell me what you were thinking when you responded to this statement*.) to assess how well they understood each item and the process through which they identify item responses. Participants were also asked for overall feedback (e.g., *Do you think any of these statements can be eliminated?*). Items that were reported as unclear or interpreted differently across participants were re‐worded, and items that were deemed unnecessary were removed. Interviews continued until the measure required no more modifications. No items were removed in this phase, but four items (items 3, 4, 8, and 15) were edited based on patient feedback.

### 3.3 Study Phase 3: EDT Validation Testing

#### 3.3.1 Demographics

During Phase 3 EDT validation testing, the research team contacted 836 patients, of whom 310 agreed to participate. Nine patients indicated they were no longer interested in being included in the study or were later deemed ineligible, leaving 301 included for data analysis. Most of the participants identified as cisgender women (*n* = 187, 62%); close to half (*n* = 147, 49%) were White, and the mean age was 47 years old (range 18–88 years). Less than half of the sample had a full‐time job (*n* = 124, 41%). Most individuals had either Medicaid (44%, *n* = 133) or Medicare (23%, *n* = 70) insurance, and most had a primary care physician (*n* = 256, 85%). See Table 1 for additional details.

**TABLE 1:** Demographics (*N* = 301).

Characteristic	*n*	%
Race^a^		
White	147	48.8
Black	110	36.5
Hispanic/Latino/x	41	13.6
Asian	6	2.0
American Indian/Alaska Native	4	1.3
Native Hawaiian/Pacific Islander	2	0.7
Other	9	3.0
Decline	4	1.3
Gender^a^		
Man	112	37.2
Woman	187	62.1
Transgender/gender nonconforming	2	0.6
Employment^a^		
Fulltime	124	41.2
Part‐time	28	9.3
Self‐employed	11	3.7
Looking for work/unemployed/student	63	20.9
Disabled	33	11.0
Retired	47	15.6
Homemaker	1	0.3
Decline	1	0.3
Insurance^a^		
Private	122	40.5
Medicare	70	23.3
Medicaid	133	44.2
No insurance	10	3.3
Unsure	4	1.3
Decline	1	0.3
Income		
< $10,000	14	4.7
$10,000–24,999	39	13
$25,000–49,999	49	16.3
$50,000–99,999	62	20.6
≥ $100,000	51	16.9
Unsure	75	24.9
Decline	11	3.7

^a^ Participants could select multiple options. Therefore numbers exceed 301.

#### 3.3.2 Exploratory Factor Analysis

Prior to the EFA, the inter‐item correlation matrix was evaluated, which indicated that item 3 was not correlated (< 0.20) with multiple items (6, 8, 9, 15) and that item 11 was highly correlated with items 12 and 14 (> 0.7). However, to ensure a data‐driven and theoretical approach to item reduction, all items were retained for the analysis.

Goodness‐of‐fit indices were compared across 1‐, 2‐, 3‐, and 4‐factor models and indicated that the 3‐ and 4‐factor structures fit the data best (Table 2). These models were not parsimonious as they included multiple cross‐loadings which violated the 40‐30‐20 rule multiple times and had a factor loading greater than 1. Additionally, these models contained factors which did not have local identification (i.e., defined by only one or two items) and factors which were not correlated with one another as would be expected given the theoretical underpinnings. Goodness‐of‐fit indices indicated that the 2‐factor structure had adequate fit according to the SRMR and CFI indices. Additionally, this structure was parsimonious with minimal cross loadings and factors which correlated (*r* = 0.71). However, item 3 did not meaningfully load onto any factors which aligned with the inter‐item correlation matrix. This indicated that item 3 was not related to the overall construct of the measure. Therefore, item 3 was removed, and another EFA was conducted which compared 1‐, 2‐, 3‐, and 4‐factor models. Goodness‐of‐fit indices indicated that the 3‐ and 4‐factor structures fit the data best while the 2‐Factor structure had an adequate fit for the data. The 3‐ and 4‐factor models were not parsimonious, violated the 40‐30‐20 rule multiple times, and contained factors that were not correlated with one another. Therefore, the 2‐factor structure was retained for the CFA. Factor 1 included the original items 1, 2, 4, 5, 9, 11, 12, 13, 14, 15 while Factor 2 included items 6, 7, 8, 10.

**TABLE 2:** Exploratory factor analysis fit indices.

	AIC	SABIC	Chi‐square	RMSEA	CFI	TLI	SRMR
1‐Factor	7955.90	7980.00	< 0.05	0.11	0.88	0.86	0.05
2‐Factor	7854.52	7886.12	< 0.05	0.10	0.92	0.89	0.05
3‐Factor	7799.31	7837.88	< 0.05	0.09	0.94	0.91	0.04
4‐Factor	7744.90	7789.90	< 0.05	0.08	0.97	0.93	0.03
*EFA without item 3*							
1‐Factor	7328.48	7350.98	< 0.05	0.11	0.90	0.88	0.05
2‐Factor	7227.03	7256.49	< 0.05	0.09	0.94	0.91	0.04
3‐Factor	7138.11	7219.00	< 0.05	0.08	0.96	0.93	0.03
4‐Factor	7151.41	7193.20	< 0.05	0.07	0.98	0.95	0.03

#### 3.3.3 Sequential Comparisons

A CFA was first conducted on the 2‐factor model. See Table 3 for goodness‐of‐fit indices of all analyses. Modification indices were reviewed and compared with the theoretical framework. These indices indicated that the model would improve if item 9 was included on the other factor, which aligned with the original purpose of the way in which the question was developed. Another CFA was conducted, and goodness of fit indices indicated an adequate fit with slightly improved AIC and SABIC values. All loadings were greater than 0.35 and all variances were between 0.10 and 0.90, which indicated adequate measure quality needed for ESEM.

**TABLE 3:** Confirmatory factor analysis goodness of fit indices.

Models	Description	*χ* ^2^	df	AIC	SABIC	CFI	TLI	RMSEA	SRMR
CFA 1	2‐factor	271.836	76	9510.836	9510.596	0.926	0.912	0.093	0.042
ESEM	2‐factor	51.428	26	7000.422	7021.313	0.984	0.972	0.057	0.023
CFA 2	2‐factor	74.678	34	7007.671	7024.277	0.974	0.966	0.063	0.029
BICFA	2‐factor	54.087	25	7005.080	7026.507	0.982	0.967	0.062	0.024

A subsequent ESEM was conducted, and goodness of fit indices indicated an adequate fit. As expected, the ESEM initially produced invalid solutions possibly due to item redundancy (e.g., factor loading > 1). Therefore, several ESEM models were run consecutively as redundant items were removed (items 4, 7, 12, and 14). Another ESEM analysis was conducted which yielded an excellent fit for the data which was compared to the goodness of fit indices of a CFA. As the differences in the goodness of fit indices were trivial between the ESEM and CFA outputs, the CFA results were retained.

#### 3.3.4 Bi‐Factor Model

A CFA Bi‐Factor Model was fit and yielded slightly improved results from the CFA (*χ* ^2^ (25) = 54.09, *p* < 0.05; AIC = 5394.43; SABIC = 5415.86; CFI = 0.98, TLI = 0.97; RMSEA = 0.06, and SRMR = 0.02). All items loaded significantly on the overall *general* factor. No items significantly loaded onto Factor 1. Two of the four items on Factor 2 loaded significantly onto the *general* factor. As these results only partially support a bi‐factor model, the two‐factor CFA model was retained as the final measure structure. The finalized 6‐item Factor 1 (i.e., new items 1, 2, 3, 8, 9, 10) was named “Comfort with Care Received” as these items centered on patient perspectives of the care they received while at the ED. The finalized 4‐item Factor 2 (i.e., new items 4, 5, 6, 7) was named “Understanding of Next Steps” as these items reflected the patients' understanding of follow‐up care that they may need. Of note, all items in Factor 2 had negative loadings. These subscales were highly correlated (*r* = −0.95). See Appendix E for bi‐factor confirmatory factor analysis results. See Table 4 for two‐factor confirmatory factor analysis results.

**TABLE 4:** Confirmatory factor analysis results.

	Factor 1	Factor 2
*I am leaving the emergency department understanding…*		
1. What medical problems I had been tested for	0.64*	
2. That “life‐threatening” or “dangerous” conditions had NOT been found	0.67*	
3. Why certain things (e.g., testing, specialist evaluation) were NOT done during my visit	0.58*	
4. How to care for myself/my symptoms in the following days		−0.76*
5. The symptoms or situations to watch for as reasons that I would need to return to the emergency department		−0.71*
*I am leaving the emergency department confident…*		
6. That I would be able to manage my symptoms in the next few days		−0.80*
7. That I would be able to complete any recommended next steps for follow up care		−0.72*
8. That my concerns were taken seriously	0.85*	
9. That I did NOT need to get a second opinion right away at a different emergency department or other care setting	0.78*	
*I am leaving the emergency department feeling…*		
10. Safe going home based on the results of emergency department visit	0.80*	

*Note:* Item factor loadings for Factor 2 were negative. The Factor 1 and Factor 2 subscales were also negatively correlated.

* *p* < 0.05.

#### 3.3.5 Reliability and Validity

Reliability analyses were conducted for the total measure and each factor. Reliability for the global score was excellent (0.93), while the reliabilities for Factors 1 and 2 were adequate (0.87 and 0.83, respectively). Bivariate correlations were conducted to evaluate convergent and discriminant validity using scales that measure similar and dissimilar constructs. Prior to assessing bivariate correlations, scores for the EDT Global measure and each subfactor were calculated. As the response options ranged between *Strongly Disagree* and *Strongly Agree*, scores on each individual item ranged between 1 and 4 and were summed to obtain a global score and scores on each subfactor. Therefore, the global score could range between 10 and 40. Scores on the 6‐item Factor, Comfort with Care Received, could range between 6 and 24 while scores on the 4‐item Factor, Understanding of Next Steps, could range between 4 and 16. See Appendix F for measure and scoring instructions. The bivariate correlation matrix (Table 5) indicated that the CTM‐3 and the CAT‐T were both significantly correlated with both factors of the EDT and the EDT Global Score. Lastly, the STAI‐state inventory and the PROMIS companionship measures were not significantly correlated with any subscale or global score of the EDT.

**TABLE 5:** Bivariate correlations.

	CTM‐3	CAT‐T	STAI	PROMIS	Factor 2	Factor 1	Global
CTM‐3	1.00						
CAT‐T	0.531*	1.00					
STAI	0.031	0.021	1.00				
PROMIS	0.107	0.035	−0.046	1.00			
Factor 2	0.643*	0.589*	0.052	0.108	1.00		
Factor 1	0.683*	0.678*	0.007	0.058	0.813*	1.00	
Global	0.701*	0.670*	0.020	0.083	0.930*	0.970*	1.00

* Significant at the < 0.01 level.

## 4 Discussion

We employed a rigorous development process to assess validity that included expert panel feedback (content validity), patient input for item refinement (process response), statistical analysis of the measure structure (structural validity), and bivariate correlations (convergent and discriminant validity). Analysis of the measure structure ultimately yielded excellent goodness of fit indices which support structural validity. The non‐significant findings between the EDT and STAI‐SF, a measure of anxiety, suggest that the EDT measures a separate, unrelated construct, indicating discriminant validity. Simply put, the EDT does not measure a patient's anxiety. This evidence further differentiates the EDT from other patient reported outcome measures (PROs), particularly as PROs are often used to capture symptoms, such as anxiety [41]. While understanding patient anxiety is important, it can place the onus of the patient experience on the patient and take emphasis away from improving the care transition's process in the ED environment. Our non‐significant correlation suggests that the EDT is capturing a more comprehensive construct of care transition regardless of a person's anxiety state. Lastly, the EDT was moderately to strongly correlated with measures of provider communication and another measure of care transition readiness which indicates convergent validity. Lastly, high internal consistency (*α* = 0.93) indicated adequate reliability. The final 10‐item 2‐factor EDT measure demonstrates preliminary evidence of content validity, response process, construct validity, and high internal consistency (i.e., reliability). To that end, this study adds a valuable tool to the patient‐reported outcomes measure literature.

This work is innovative in multiple ways. Care transitions home from the ED have been identified as a period of increased risk for patient safety concerns. Diagnostic challenges, such as diagnostic uncertainty, further increase the potential for patient safety concerns. Thus, patients discharged with diagnostic uncertainty may be at increased risk for patient safety events, indicating a need for improved care transitions. Yet, while over one third of patients are discharged from the ED with diagnostic uncertainty, no measurement tools to assess care transitions have been designed that are inclusive of patient experiences in the setting of diagnostic uncertainty. Importantly, our discriminant validity findings may suggest that solely assessing a patient's uncertainty (through the U‐scale) or anxiety (through the STAI) does not sufficiently capture patients' perceptions of their quality‐of‐care transitions upon completion of an ED visit. Thus, the EDT is differentiated from other scales, such as the U‐Scale, which considers the patient's *uncertainty state* related to diagnosis as opposed to their perceived readiness for care transition. Inclusion of both measures in future studies may yield rich information on patients' perceptions of both their uncertainty diagnosis and the care transition process.

Further, while most intervention or clinical pathways focus on a specific disease state or condition, the EDT is disease and diagnosis agnostic, thus supporting work focused on improving the patient experience across disease states. This lack of disease focus enables the use of this tool not only for the use in “diagnostic uncertainty” studies, but also in other contexts where the patient is discharged on a diagnostic pathway such as rapid outpatient chest pain and headache or even with a diagnosis. Developing disease agnostic measures that can be used to understand patients' experiences with transitions of care may help facilitate the development of high‐quality clinical trials aimed at improving care transitions for populations at increased risk for patient safety events.

While this work began with the purpose of filling a measurement void for the many patients who leave the ED with diagnostic uncertainty, the final measure that emerged through this development process has items that are relevant regardless of receiving a definitive or uncertain diagnosis. Thus, instead of being specific to uncertainty, this measure is inclusive of it. While we have validated the EDT among the population of patients with diagnostic uncertainty, further work is needed to validate it more broadly among the rest of the ED population.

### 4.1 Limitations

This study has several limitations. First, the participant pool was recruited from only one health enterprise. This may reduce the generalizability of the measure to other geographic regions and care settings that have different clinic‐level guidelines for care transitions and managing discharges for patients with diagnostic uncertainty. These differences may contribute to different item‐level factor loadings, which consequently will impact the internal structure of the measure. While the inclusion of patients from 12 hospitals across 9 counties and 2 states should minimize this limitation, future studies including populations from different health contexts can improve generalizability. Additionally, this study used the CAT‐T, which has been used in many previous studies to assess patient perspectives; however, it has limited evidence of its validity [42, 43]. Thus, inferences made based on its correlation with the EDT must be interpreted with caution, and additional studies need to be done to continue to examine the validity of the EDT. Also, the measure was designed with a focus on measuring transitions for patients with diagnostic uncertainty, and only patients with symptom‐based diagnoses were included in the validation study. Therefore, the EDT has not been validated in a general ED population. However, patients with diagnostic uncertainty represent a very heterogeneous population both demographically and medically, with complaints spanning many body systems.

As with any newly developed measure, additional research should also be conducted to evaluate the measure's properties in a different sample, such as reliability, convergent, and discriminant validity. Future studies should also assess other aspects of validity including predictive validity to understand pathways between care transitions and health outcomes and healthcare utilization. Additionally, this study did not assess measurement invariance, so we are unable to conclude how this measure performs in diverse populations who may have different lived experiences based on identities such as class, gender, race, and ethnicity. Lastly, this measure is written in English and does not have a translated version. While this is unfortunately typical in many PROs and other self‐report measures, we recognize that this limits our ability to test the EDT in non‐English speaking populations and assess transitions of care for certain vulnerable groups.

## 5 Conclusions

In conclusion, we evaluated evidence relevant to content, discriminant, and convergent validity, and evidence supported the use of the Emergency Department Transitions measure to assess quality of care transitions from the ED for patients with diagnostic uncertainty. Diagnostic uncertainty and the provision of symptom‐based diagnoses are ubiquitous in emergency care, yet previously there was a lack of any PRO to measure the patient experience of the care transition home. This newly created tool can be used in future work to both better understand patients' readiness for transitions of care home in the current state as well as quantify the impact of interventions focused on improving quality of care in the ED and during the transition home. Further, this tool has the potential to evaluate quality of care delivery by capturing patient perspectives, specifically related to care transitions, on their care in the ED. Thus, this tool adds to the growing field of PROs and is especially complementary to the existing PRO measure toolbox in the field of emergency medicine.

## Author Contributions

K.L.R., R.H., and DM contributed to study concept and design. S.B., J.L., A.G., M.W., P.O., and M.T. contributed to acquisition of the data. S.B., D.M., R.H., and K.L.R. contributed to analysis and interpretation of the data. S.B., J.L., and K.L.R. drafted the manuscript. All authors critically reviewed the manuscript. S.B. and R.H. contributed to statistical expertise.

## Funding

The authors have nothing to report.

## Conflicts of Interest

Drs. Rising and McCarthy receive funding from AHRQ focused on diagnostic uncertainty. None of those funds were used for this project.

## Data Availability

The data that support the findings of this study are available on request from the corresponding author. The data are not publicly available due to privacy or ethical restrictions.

## Appendix A

The following list of patient complaints was determined to be diagnostic uncertainty:

**Table:**

ICD code	Symptoms
R07	
R07.1	Chest pain on breathing
R07.8	Other chest pain
R07.81	Pleurodynia
R07.82	Intercostal pain
R07.89	Other chest pain
R07.9	Chest pain, unspecific
R51	
R51.0	Headache with orthostatic component, not elsewhere classified
R51.9	Headache, unspecified
R10	
R10.1	Pain localized to upper abdomen
R10.10	Upper abdominal pain, unspecified
R10.11	Right upper quadrant pain
R10.12	Left upper quadrant pain
R10.13	Epigastric pain
R10.2	Pelvic and perineal pain
R10.3	Pain localized to other parts of lower abdomen
R10.30	Lower abdominal pain, unspecified
R10.31	Right lower quadrant pain
R10.32	Left lower quadrant pain
R10.33	Periumbilical pain
R10.8	Other abdominal pain
R10.81	Abdominal tenderness
R10.812	Right upper quadrant abdominal tenderness
R10.813	Left upper quadrant abdominal tenderness
R10.814	Left lower quadrant abdominal tenderness
R10.815	Periumbilical abdominal tenderness
R10.816	Epigastric abdominal tenderness
R10.817	Generalized abdominal tenderness
R10.819	Generalized abdominal tenderness, unspecified site
R10.82	Rebound abdominal tenderness
R10.821	Right upper quadrant rebound abdominal tenderness
R10.822	Left upper quadrant rebound abdominal tenderness
R10.823	Right lower quadrant rebound abdominal tenderness
R10.824	Left lower quadrant rebound abdominal tenderness
R10.825	Periumbilical rebound abdominal tenderness
R10.826	Epigastric rebound abdominal tenderness
R10.827	Generalized rebound abdominal tenderness
R10.829	Generalized rebound abdominal tenderness, unspecified site
R10.83	Colic
R10.84	Generalized abdominal pain
R10.9	Unspecified abdominal pain
R06	
R06.0	Dyspnea
R06.00	Dyspnea, unspecified
R06.02	Shortness of breath
R06.09	Other forms of dyspnea
R05	
R05.1	Acute cough
R05.2	Subacute cough
R05.8	Other specified cough
R05.9	Cough, unspecified
R21	Rash and other nonspecific skin eruption
R42	Dizziness and giddiness

## Appendix B Measures Used for Item Generation

**Table:**

Scale name	Brief description of measure	Target population	Validity and reliability evidence	Items used to generate new items	Example of items
Uncertainty Scale (U‐Scale)	The U‐Scale assesses a patient's state of uncertainty related to having symptoms at a discrete point in time as opposed to capturing a more comprehensive assessment of readiness for a care transition home	Patients at the time of ED discharge	Reliability—High internal consistency (*α* = 0.93) [22] Content validity—Robust procedures, Group Content Mapping, were used to identify important domains [23] Predictive validity—Scores on the Decision to Seek Care and Treatment Quality subscales correlated with higher odds of 30‐day ED returns [21]	2, 3, 4, 5, 6, 7, 8, 9, 11, 12, 13, 14, 16, 17, 18, 19, 20, 21, 22, 23, 24, 28, 29, 30	“I have the knowledge and ability to treat my symptoms.” “I often feel like my doctors don't give me enough information about my tests results.”
Uncertainty Communication Checklist (UCC)	The UCC is a clinician‐facing tool and was developed as a conversational approach to promote more effective ED discharge conversations between patients and clinicians when patients are discharged with diagnostic uncertainty	ED Physicians	Content validity—physicians reviewed and ranked items, indicating they were important to incorporate in communication with patients [25]	3, 4, 5, 6, 7, 9, 10, 12, 13, 14, 15, 16, 17, 18, 19	“Clearly state that either ‘life‐threatening’ or ‘dangerous’ conditions have not been found.” “Discuss next tests that are needed, if any.”
Short Form Care Transitions Measure (CTM‐3)	The CTM was developed as a useful measure to capture the quality of hospital‐to‐home care transitions to aid quality improvement	General patient population	Predictive validity—Higher scores correlated with decreased likelihood of returning to the ED [13] Predictive validity—Higher scores correlated with increased likelihood of taking medications as prescribed [13]	1, 2	“The hospital staff took my preferences and those of my family or caregiver into account in deciding what my health care needs would be when I left the hospital.” “When I left the hospital, I had a good understanding of the things I was responsible for in managing my health.”

## Appendix C Expert Panelists

**Table:**

Expert panel member	Domains of expertise
Paul Han, MD, MA, MPH	Uncertainty in medicine, risk communication
Sara Malone, PhD, MSW	Measure development, Emergency Department social work
Hardeep Singh, MD, MPH	Understanding and reducing diagnostic errors, Diagnostic Safety
Margaret Samuels‐Kalow, MD, MPhil, MSHP	Emergency Medicine, quality and equity of care, health literacy, health services research
Manish Shah, MD, MPH	Emergency Medicine, transitions of care

## Appendix D Definitions

1. Exploratory factor analysis (EFA) – An EFA is an exploratory analysis that looks for patterns in data that can be grouped together and reflects a specific construct. These data are grouped together based on the strength of the item‐level correlations with the underlying factors. As this is an exploratory analysis, items are allowed to load on multiple factors.
2. Confirmatory factor analysis (CFA)—A CFA is a confirmatory analysis that examines how well a model based on a previous EFA, research, or theory fits the data. As this is a confirmatory analysis, items are only allowed to load on their hypothesized factor and are constrained to 0 on other factors. Goodness of fit indices are used to determine whether the model fits the data better than a null hypothesis or a predicted model.
3. Exploratory Structural Equation Modeling (ESEM)—An ESEM analysis blends EFA and CFA procedures. An ESEM analysis allows items to cross‐load while imposing a structure. Fit indices are also used to determine if the proposed structure, or model, fits the data well.
4. Absolute fit indices—These fit indices compare the observed relationships that the actual data shows with the implied, or predicted, relationships constructed in the model. Absolute fit indices included in this study include SRMR, RMSEA, AIC, SABIC, and *χ* ^2^. A non‐significant *χ* ^2^ *p*‐value > 0.05 indicates adequate fit. Values < 0.08 for the root‐mean‐square‐error of approximation (RMSEA) and values < 0.05 for the standardized root‐mean‐square residual (SRMR) indicate adequate fit. Values closer to 0 indicate a better fit for AIC and SABIC. AIC and SABIC are also used to compare model fit. A difference of 10 indicates a meaningful difference between models.
5. Incremental fit indices—These fit indices compare whether the proposed model fits the data better when compared to the null model which assumes all variables are unrelated. These fit indices provide information on how well the proposed model improves fit from the “worst‐case” model (i.e., null model). Incremental fit indices used in this study include the CFI and TLI. Values > 0.90 and > 0.95 for the comparative fit index (CFI) and the Tucker‐Lewis model index (TLI) indicated adequate and excellent fit, respectively.
6. 40‐30‐20 Rule—This rule helps researchers identify a factor structure that is easily interpretable and can be meaningfully used in the context of measure development. This rule states that items should load onto a factor with at least a 0.40 coefficient, should have a double‐loading of < 0.30, and the difference between each double‐loading should be at least 0.20.
7. Parsimony—Parsimony refers to simplicity in a statistical model. Complex data becomes difficult to use as it is harder to interpret and also harder to meaningfully translate into real‐world settings. A parsimonious solution is not overly complex, has as few parameters as are needed, and can be easily interpreted. Thus, parsimony must be taken into account when interpreting data, especially when developing measures. Overly complex measures are cumbersome for patients and become too difficult to meaningfully use in studies.
8. Rotations—EFA and ESEM both utilize rotations. These rotations refer to the computer program rotating data in a way which makes it easier to interpret while maintaining the same mathematical equation. Oblique rotations are used when factors are hypothesized to correlate. In this study, we used Geomin, which is an oblique rotation for the EFA and a target rotation for the ESEM.

## Appendix E Bi‐Factor Confirmatory Factor Analysis Results

**Table:**

	G Factor	Factor 1	Factor 2
*I am leaving the emergency department understanding…*			
1. What medical problems I had been tested for	0.64*	0.46	
2. That “life‐threatening” or “dangerous” conditions had NOT been found	0.67*	0.09	
3. Why certain things (e.g., testing, specialist evaluation) were NOT done during my visit	0.59*	−0.14	
4. How to care for myself/my symptoms in the following days	0.71*		0.45*
5. The symptoms or situations to watch for as reasons that I would need to return to the emergency department	0.67*		0.20
*I am leaving the emergency department confident…*			
6. That I would be able to manage my symptoms in the next few days	0.77*		0.10*
7. That I would be able to complete any recommended next steps for follow up care	0.72*		0.09
8. That my concerns were taken seriously	0.85*	−0.01	
9. That I did NOT need to get a second opinion right away at a different emergency department or other care setting	0.77*	0.12	
*I am leaving the emergency department feeling…*			
10. Safe going home based on the results of emergency department visit	0.81*	−0.11	

* *p* < 0.05.

## Appendix F EDT Measure and Scoring Instructions

**Table:**

	Strongly Disagree	Disagree	Agree	Strongly Agree
*I am leaving the emergency department understanding…*				
1. What medical problems I had been tested for	Strongly Disagree	Disagree	Agree	Strongly Agree
2. That “life‐threatening” or “dangerous” conditions had NOT been found	Strongly Disagree	Disagree	Agree	Strongly Agree
3. Why certain things (e.g., testing, specialist evaluation) were NOT done during my visit	Strongly Disagree	Disagree	Agree	Strongly Agree
4. How to care for myself/my symptoms in the following days	Strongly Disagree	Disagree	Agree	Strongly Agree
5. The symptoms or situations to watch for as reasons that I would need to return to the emergency department	Strongly Disagree	Disagree	Agree	Strongly Agree
*I am leaving the emergency department confident…*				
6. That I would be able to mange my symptoms in the next few days	Strongly Disagree	Disagree	Agree	Strongly Agree
7. That I would be able to complete any recommended next steps for follow up care	Strongly Disagree	Disagree	Agree	Strongly Agree
8. That my concerns were taken seriously	Strongly Disagree	Disagree	Agree	Strongly Agree
9. That I did NOT need to get a second opinion right away at a different emergency department or other care setting	Strongly Disagree	Disagree	Agree	Strongly Agree
*I am leaving the emergency department feeling…*				
10. Safe going home based on the results of emergency department visit	Strongly Disagree	Disagree	Agree	Strongly Agree

*Note:* Scoring Instructions for total score: Each individual item is scored from 1 to 4 (i.e., *Strongly Disagree =* 1, *Disagree =* 2, *Agree =* 3, *Strongly Agree =* 4). To obtain the score for the 6‐item factor, *Comfort with Care Received*, add up items 1, 2, 3, 8, 9, and 10 (i.e., possible range between 6 and 24). To obtain the score for the 4‐item factor, *Understanding of Next Steps*, add up items 4, 5, 6, and 7 (i.e., possible range 4–16). Add all items together to obtain a global score (i.e., possible range 10–40).
